# Prevalence of diminished kidney function in a representative sample of middle and older age adults in the Irish population

**DOI:** 10.1186/1471-2369-13-144

**Published:** 2012-11-02

**Authors:** Gemma M Browne, Joseph A Eustace, Anthony P Fitzgerald, Jennifer E Lutomski, Ivan J Perry

**Affiliations:** 1 Department of Epidemiology and Public Health, University College Cork, Western Gateway Building, Cork, Ireland; 2 Department of Medicine, Mercy University Hospital, Cork, Ireland; 3 Clinical Research Facility, University College Cork, Cork, Ireland; 4 Department of Nephrology, Cardiac Renal Centre, Cork University Hospital, Cork, Ireland

**Keywords:** Chronic kidney disease, Glomerular filtration rate, Albuminuria, Population survey

## Abstract

**Background:**

The prevalence of chronic kidney disease (CKD) using available estimating equations with the Republic of Ireland is unknown.

**Methods:**

A randomly selected population based cross-sectional study of 1,098 adults aged 45 years and older was conducted using data from the 2007 Survey of Lifestyle, Attitudes and Nutrition (SLÁN). Estimated Glomerular Filtration Rate (eGFR) was calculated from a single IDMS aligned serum creatinine using the CKD-EPI and the MDRD equations, and albumin to creatinine ratio was based on a single random urine sample.

**Results:**

The sample clinical characteristics and demography was similar to middle and older age adults in the general Irish population, though with an underrepresentation of subjects >75 years and of males. All results are based on subjects with available blood and urine samples. Applying weighting to obtain survey based population estimates, using Irish population census data, the estimated weighted prevalence of CKD-EPI eGFR<60 mL/min/1.73m^2^ was 11.6%, (95% confidence interval; 9.0, 14.2%), 12.0% ( 9.0, 14.2%) of men and 11.2% (7.3, 15.2%) of women. Unweighted prevalence estimates were similar at 11.8% (9.9, 13.8%). Albuminuria increased with lower CKD-EPI eGFR category. 10.1% of all subjects had albuminuria and an eGFR≥60 mL/min/1.73 m^2^ giving an overall weighted estimated prevalence of National Kidney Foundation (NKF) defined CKD 21.3% (18.0, 24.6%), with the unadjusted estimate of 21.9% (19.5, 24.4%). MDRD related estimates for eGFR <60 mL/min/1.73 m^2^, and NFK defined CKD were higher than CKD-EPI and differences were greater in younger and female subjects.

**Conclusions:**

CKD is highly prevalent in middle and older aged adults within the Republic of Ireland. In this population, there is poor agreement between CKD-EPI and MDRD equations especially at higher GFRs. CKD is associated with lower educational status and poor self rated health.

## Background

Chronic kidney disease (CKD) is a common condition that irrespective of aetiology results in a wide range of complications including hypertension, hyperparathyroidism, anaemia, vascular calcification and accelerated cardiovascular disease [1,2]. The symptoms associated with CKD are vague and are often attributed to age-related frailty; as a result CKD often remains undiagnosed until the condition is advanced.

The diagnosis of CKD is ideally established by measurement of the glomerular filtration rate (GFR). As in other population based surveys, the measurement of ^125^ I-iothalamate [3] which is recognised as the gold standard method to determine GFR, is costly, invasive and impractical in larger studies. In addition, timed urine collections suffer from inaccuracy especially in community dwelling ambulatory subjects and isolated serum creatinine levels are difficult to interpret, due to the influence of body habitus and age related reduction in muscle mass. This has resulted in GFR estimating equations entering widespread clinical use. To date, the best validated and widely used estimation equation has been the Modification of Diet in Renal Disease (MDRD) equation [4-6]. Across laboratories in Ireland and in the UK, the MDRD equation is used routinely to estimate GFR. An alternative equation, the Chronic Kidney Disease Epidemiology Collaboration (CKD-EPI) equation [7], has been recently developed by a NIH consortium based on pooled data with a better representation of subjects with normal renal function. It is expected that this equation should improve the accuracy and validity of eGFR when used in population based screening relative to the MDRD equation. As with other causes of chronic disease, social and economic deprivation has been shown to influence the development and progression of CKD, in individual subjects and in residential communities [8-10].

To date there has been no population based estimates of the prevalence of CKD in Ireland. We therefore conducted the following analysis using data collected during a national representative cross-sectional survey of the Irish population in order:

(i). To estimate the prevalence of diminished eGFR in middle and older aged adults in the Republic of Ireland and overall prevalence weighted for any underrepresented groups

(ii). To compare the effect of using the more appropriate derived CKD-EPI equation as compared to the established MDRD equation, which to date remains in widespread clinical use.

(iii). To explore the associations of CKD with self rated health, education, and socio-economic group and population based diabetes mellitus in Ireland.

## Methods

A population-based cross-sectional analysis of renal function in adults aged 45 years or older was conducted using data from the 2007 Survey of Lifestyle, Attitudes and Nutrition (SLÁN), which is the most recent of a series of nationally representative health surveys in the Republic of Ireland. Full details on survey methods have been presented elsewhere [11]. A random sample of the Irish population was obtained using as its sampling frame ‘The Irish GeoDirectory’, listing all residential, non commercial addresses in the Republic of Ireland, compiled by the Irish Postal service. Non-institutionalized adults aged 18 years and older were recruited using multi-stage sampling with a known probability of selection for each dwelling. The primary sampling units were based on town land aggregates, with stratification by area characteristics and region, 400 such clusters were systematically selected using a random national starting point. Forty-six addresses were then systematically selected using a random starting point within each cluster. One adult was selected from each house as the subject with the nearest birthday on the study visit day. Overall, 10,364 participants (62% of those invited to participate) completed the extensive health and lifestyle questionnaire. A 25% random subgroup of the study participants who were 45 years or older were selected by inviting all such subjects within randomly selecting clusters to further undergo a comprehensive physical examination and basic laboratory testing. The surveys were confined to this age group, to allow comparison with earlier Irish based surveys (SLAN 1998, SLAN 2002), and as resources were not available to sample all age groups. The response rate to the physical examination was 66% with 179 subjects unable to participate and 613 subjects declining to participate. 47 subjects who participated in the physical examination component did not provide serum specimens, in addition 62 participating subjects were unable to provide an adequate urine specimen, resulting in a sample size for the albuminuria data of 1,098. In order to correctly interpret results based on eGFR and albuminuria only results based on 1098 subjects are presented.

For comparative purposes, an eGFR was calculated from a single, presumed stable, serum creatinine measurement using both the MDRD and the CKD-EPI regression equations as previously published [6,7]. Both equations measure eGFR in mL/min/1.73 m^2^ of body surface area (BSA). We categorised renal function based on the National Kidney Foundation - Kidney Disease Outcomes Quality Initiative (NKF-K/DOQI) classification into G1 for GFR ≥90, G2 for 60–89, G3A for 45–59, G3B for 30–44, G4 for 15–29 and G5 for <15 mL/min/1.73 m^2^ , respectively. This has been recommended in the recent literature to allow direct staging of eGFR, in addition to classification of CKD status [12,13]. In accordance with the K/DOQI definition an eGFR<60 mL/min/1.73 m^2^ was diagnostic of CKD, while for an eGFR≥60 mL/min/1.73 m^2^, additional evidence of renal damage is necessary, for which we exclusively used the presence of an albumin to creatinine ratio of >30 mg/g [12,13]. All specimens were collected in a non-fasting state. Serum creatinine was measured using the kinetic rate Jaffe method, and was IDMS aligned. Urine albumin was measured using bromcresol green method. Urinary creatinine concentration was measured on the same single void specimen and expressed as an albumin to creatinine ratio to adjust for differences in urinary concentration.

Household social class (SC) categories were derived from occupational status using the European socio-economic status *(SES)* classification system [14], and were divided into higher (professional and managerial) social classes (SC 1–2), non-manual and skilled manual categories (SC 3–4), and semi-skilled and unskilled occupational categories (SC 5–6) and unclassifiable on the basis of present or last occupation. Education is described as complete primary-level education only or with incomplete second-level education, complete second-level education, and some form of third-level education including Diplomas, College and post-graduate degrees. Smokers were identified as participants who reported smoking some days to every day, and former smokers reported smoking at least 100 cigarettes in the past and non smokers. Based on the International Physical Activity Questionnaire (IPAQ) score [15,16], participants’ activity levels were classified as light (<5,000 steps per day) and moderate to heavy (>5,000 steps per day). The presence of diabetes mellitus was assessed combining three methods, two questions relating to the diagnosis of diabetes, and / or the presence of diabetic medications including insulin and measurement of haemoglobin A1c >6.5% [17].

### Statistical considerations

The distribution of CKD-EPI eGFR within the sample with available serum creatinine was calculated using the NKF-K/DOQI CKD categories as defined above. G4 and G5 were denoted as a combined stage G4-G5 due to small numbers. GFR was also dichotomized into ≥60 mL/min and <60 mL/min. To extrapolate from the SLAN sample, an estimated overall population prevalence of CKD in Ireland, weights were applied which were developed by the SLAN study consortium and the Economic and Social Research Institute, Ireland. The SLAN Physical Examination sample [11] was weighted to closely approximate the Irish population census 2006 figures for gender, age, marital status, education, occupation, household size and ethnicity. Missing blood samples occurred in 3.8% of the study participants and missed urine and blood samples occurred in 9%. Age, gender and SES were similar in the entire group (N=1207) to those with blood and urine samples (N=1098) which suggests the missing data occurred on a random basis. We present the overall population prevalence as the weighted estimates [18]. The mean (95% CI) levels of agreement between the MDRD and CKD-EPI was calculated for overall study group and separately by severity of CKD, age and gender. Bland Altman plots graphically demonstrate differences and bias across the two methods in both males and females and age categories. Differences in average eGFR between methods were examined using independent sample t tests; significance level was defined as 0.05.

We examined associations between NKF-K/DOQI defined CKD with poor self-rated health, lower SES and lower educational state using a chi squared test. For the purposes of this analysis, primary school education only and incomplete second level education were merged as a single category. In addition, unclassified employment status was included with SES V. Multiple logistic regression was used to examine the association between CKD and self rated health, SES and educational status adjusted for age and gender and presented as odds ratio (OR). All statistical analyses were conducted using STATA (Version 11.0).

## Results

### Demography of the study population

The SLAN study population with available serum creatinine and proteinuria measurements (N=1098) were broadly comparable to the parent SLAN population (N=5147), though slightly younger, better educated and with a higher SES (Table 1). The age distribution of the study population, both overall and for the laboratory sample, is similar to 2006 Republic of Ireland National census data [19], although subjects over 75 years were underrepresented in the sample, (7.5% compared to 14.7%, national census data) and women were overrepresented (55.1% compared to 51.8%, national census data). The median serum creatinine in men was 89.7 micromol/L, with a slightly higher mean (SD) 93.3 micromol/L (30.2) due to presence of a small number of men with high serum creatinine levels. Mean (SD) creatinine level in women was 71.2(14.3) micromol/L with a median of 69.0 micromol/L. The mean (SD) CKD-EPI eGFR (mL/min/1.73 m^2^) was 81.0(16.7); 79.4(17.0) in men and 82.2(16.5) in women.

**Table 1:** Socio-demographic and physiological characteristics, Republic of Ireland, 2007 comparing study subjects with serum biochemical measurements and subjects with Albumin Creatinine ratio results (n=1,098) compared to SLAN 07 subjects of 45 years and older (N=5,147)

	**N=1098 N (%)**	**N=5147 SLAN07 (N%)**
**Sex**		
Male	493(44.9)	2202 (42.8)
Female	605 (55.1)	2945 (57.2)
**Age group (years)**		
45-54	422 (38.4)	1717 (33.4)
55-64	325 (29.6)	1461(28.4)
65-74	269 (24.5)	1150 (22.3)
75+	82 (7.5)	819 (15.9)
**S-E Group**		
SEG 1-2	425 (38.7)	1550 (30.1)
SEG 3-4	368 (33.5)	1943 (37.7)
SEG 5	250 (22.8)	985 (19.1)
*Unclassified*	55 (5.01)	334 (6.4)
**Education**		
Primary or less	223 (20.3)	1600(31.1)
Incomplete 2nd	252 (23.0)	1171 (22.8)
Completed 2nd	221 (20.1)	1051 (20.4)
College	402 (36.6)	1325 (25.7)
**Smoking status**^§^		
Never	529 (48.8)	2,872 (56.5)
Former	356 (32.8)	1,349 (26.5)
Current	199 (18.4)	1,061 (20.9)
**Self-rated health**^§^		
Excellent/very good/good	903 (82.5)	3984 (77.6)
Fair/poor	192 (17.5)	1148 (22.4)
**Physical Activity**^§^		
Moderate/heavy activity	664 (70.7)	3373 (66.3)
Light activity	275 (29.3)	1714 (33.7)
**Diabetes Mellitus**	86 (7.8)	N/A
Blood Urea (mmol/l)*		
Males	6.4 (2.1)	N/A
Females	5.6 (1.5)	N/A
Haemoglobin (g/dl)*		
Males	15 (1.3)	N/A
Females	14 (1.0)	N/A
Blood Creatinine (micromol/l)*		
Males	93.3 (30.2)	N/A
Females	71.2 (14.3)	N/A
Urine Albumin/Creatinine (mg/g)*	23.4(118.9)	
ACR >20	254 (23.1)	N/A
ACR >30	145 (13.2)	N/A

* Blood Haemoglobin, Urea, Creatinine and Urine Albumin/Creatinine (mg/g) report Mean values (Standard Deviation).

^§^ Variables may not add up to total N due to missing data.

### The prevalence of diminished renal function in Irish middle and older aged adults

The distribution of CKD-EPI eGFR by stage is shown in Table 2. The percentage (95% CI) of participants with an eGFR<60mL/min was 11.8% (9.9, 13.8%) of study subjects, with 9.2% in stage G3A, 2.3% in G3B and less than half of a percent in G4/G5. The percentage of male and female participants with an eGFR <60 mL/min was 12.6% (9.6, 15.5%) and 11.2% (8.7, 13.8%) respectively. The estimated weighted prevalence of CKD-EPI eGFR <60 mL/min/1.73m^2^ was 11.6% [8] (9.0, 14.2%), 12.0% (8.7, 15.3%) of men, and 11.2%, (7.3, 15.1%) of women, so weighting had minimal impact on estimated prevalence.

**Table 2:** Prevalence (95% Confidence Interval) of GFR Stages by MDRD and CKD-EPI equations, based on subjects with available serum creatinine and urine (N=1,098) in a randomly selected representative sample of the middle and older Irish population

	**GFR Stage % (95% CI) Prevalence estimates N=1098**	**GFR Stage% (95% CI) Prevalence estimates N=1098**
	**MDRD**	**CKD-EPI**
**GFR1**	**19.8%** (17.4%, 22.1%)	**34.4%** (31.6%, 37.2%)
**GFR 2**	**65.4%** (62.6%, 68.2%)	**53.8%** (50.8%, 56.7%)
**GFR3A**	**11.8%** (9.9%, 13.7%)	**9.2%** (7.5%, 10.9%)
**GFR3B**	**2.7%** (1.8%, 3.7%)	**2.3%** (1.5%, 3.3%)
**GFR 4-5**	**0.36%** (0.07%, 0.7%)	**0.36%** (0.07%, 0.7%)
**Total eGFR<60**	**14.9% (12.8%, 17%)**	**11.8% (9.9, 13.8)**
**Population Estimate (Weighted) eGFR<60**	**15.7% (12.7%, 18.7%)**	**11.6% (9.0%, 14.2%)**
**POPULATION PREVALENCE ESTIMATES OF CKD**		
**CKD STAGES**	**MDRD**	**CKD EPI**
**CKD Stage 1**	**2.7%** (1.9%, 3.9%)	**3.8%** (2.8%, 5.1%)
**CKD Stage 2**	**7.2%** (5.7%, 8.9%)	**6.3%** (4.9%, 7.9%)
**CKD Stage 3A**	**11.8%** (9.9%, 13.8%)	**9.2%** (7.5%, 10.9%)
**CKD Stage 3B**	**2.7%** (1.8%, 3.7%)	**2.3%** (1.5%, 3.3%)
**CKD Stage 4-5**	**0.36%** (0.07%, 0.7%)	**0.36%** (0.07%, 0.7%)
**Total**	**24.8% (22.2%, 27.3%)**	**21.9% (19.5%, 24.4%)**
**Population Estimate CKD (Weighted)**	**25.1% (21.5%, 28.6%)**	**21.3% (18%, 24.6%)**

Population prevalence is estimated using both methods and inclusive of ACR>30mg/g for CKD Stage 1 and 2, using National Kidney Foundation criteria.

### The association of estimated GFR stages with albuminuria

Albuminuria was present in 13.2% (11.2, 15.2%) of the overall study sample. The proportion of subjects with albuminuria increased with GFR stage using the CKD-EPI equation, and more than doubled from 11.1% in G1 and 11.7% in G2 to 23.9% for G3. All subjects with G4-5 had at least microalbuminuria.

### NKF diagnosis of chronic kidney disease in the Irish population

Overall 11.8% of subjects had an eGFR<60 mL/min/1.73 m^2^, 10.1% of all subjects had albuminuria and an eGFR ≥60 mL/min/1.73m^2^, meeting NKF diagnostic criterion of CKD, thereby giving an overall study prevalence of CKD of 21.9%, (19.5%, 24.4%), with an overall weighted estimated prevalence of National Kidney Foundation (NKF) defined CKD 21.3% (18.0%, 24.6%), Table 2. The prevalence of reduced eGFR increased sharply with age.

### Comparison between eGFR methods

The mean (95% CI) difference in eGFR by CKD-EPI relative to MDRD was 4.9 mL/min/1.73 m^2^ (4.5, 5.2 mL/min/1.73 m^2^) for eGFR≥60 and substantially less 1.1 (0.8, 1.5 mL/min/1.73 m^2^) for eGFR<60 mL/min/1.73 m^2^, (Table 3).

**Table 3:** **Mean (95% CI) difference between the CKD-EPI and MDRD equation estimates (defined as CKD-EPI – MDRD*), *stratified by level of renal function using CKD–EPI equation as reference, age (<65 years compared to ≥65 years) and gender N (Males:%), in a randomly selected representative sample of middle and older aged Irish population**

**CKD-EPI eGFR≥60ml/min**^1^			
	**All**	**<65 years**	**≥65 years**
	**N=968**	**N=716**	**N=252**
	**Males: 46%**	**Males: 42%**	**Males: 52%**
	**Females: 54%**	**Females: 58%**	**Females: 48%**
**All**	4.9 (4.5, 5.2) ^**1**^	6.4 (6.0, 6.8) ^**2**^	0.6 (−0.2, 1. 4) ^**3**^
**Males**	3.43 (2.9, 3.9)	5.1 (4.6, 5.6)	-.0.3 (−1.4, 0.9)
**Females**	6.03 (5.5, 6.6)	7.4 (6.8, 8.0)	1.7 (0.4, 2.7)
**p value**^**4**^	***0.0001***	***0.0001***	***0.025***
**CKD-EPI eGFR <60ml/min**^**1**^			
	**All**	**<65 years**	**≥65 years**
	**N=130**	**N=31**	**N=99**
	**Males: 50%**	**Males: 35%**	**Males: 52%**
	**Females: 50%**	**Females: 65%**	**Females: 48%**
**All**	1.1 (0.8, 1.5) ^**1**^	3.7 (3.3, 4.1) ^**2**^	0.3 (0.03,0.6) ^**3**^
**Males**	0.2 (−0.2, 0.7)	3.3 (2.6, 4.0)	−0.4 (−0.8, -0.1)
**Females**	1.7 (1.3, 2.1)	3.9 (3.4, 4.4)	1.2 (0.8, 1.5)
**p value**^**4**^	***0.0001***	***0.16***	***0.0001***

^1^p values for comparisons across GFR strata for all subjects p=0.0001.

^2^p values for comparisons across GFR strata for all subjects <65 years: p = 0.007.

^3^p values for comparisons across GFR strata for all subjects >=65 years p=0.68.

^4^p value for comparisons across gender within GFR strata, and age groups.

**Figure 1:**
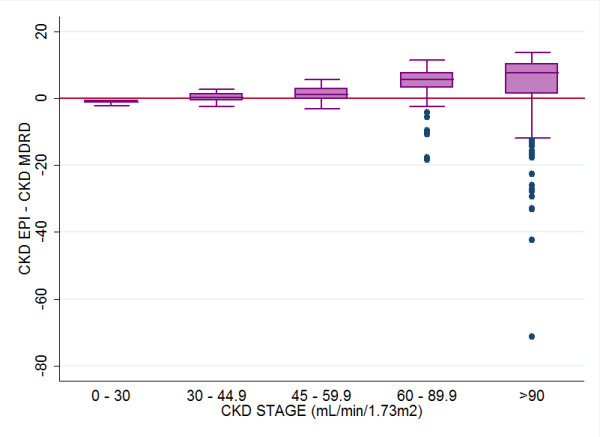
Comparison of eGFR calculated by CKD-EPI and MDRD equations (CKD-EPI − MDRD) using a box plot with non parametric IQR and range across each eGFR stage, illustrating line of agreement at CKD-EPI − MDRD=0.

Differences between the two methods by age and gender are shown in Table 3. CKD-EPI formula resulted in higher mean eGFR readings especially in younger subjects and in women. However both estimating equations agree more closely in older subjects at all levels of eGFR. While the percentage of participants calculated as having eGFR≥60 is similar using either equation (88.2% and 85.2%) within this group over half as many subjects (34.4% vs. 19.8%) are defined as having completely normal (G1) rather than slightly diminished (G2) eGFR with the CKD-EPI equation (Table 2). In addition, the MDRD estimates also have substantially more high extreme values within stage G1 than the CKD-EPI formula, values at a level that are clinically improbable. The highest eGFR (99th centile) using the MDRD equation was 195 mL/min (124.6 ml/min) versus 123.5 mL/min (108.8 mL/min) using CKD-EPI. The median and range of difference in eGFR method at eGFR stages are illustrated in Figure 1.

**Figure 2:**
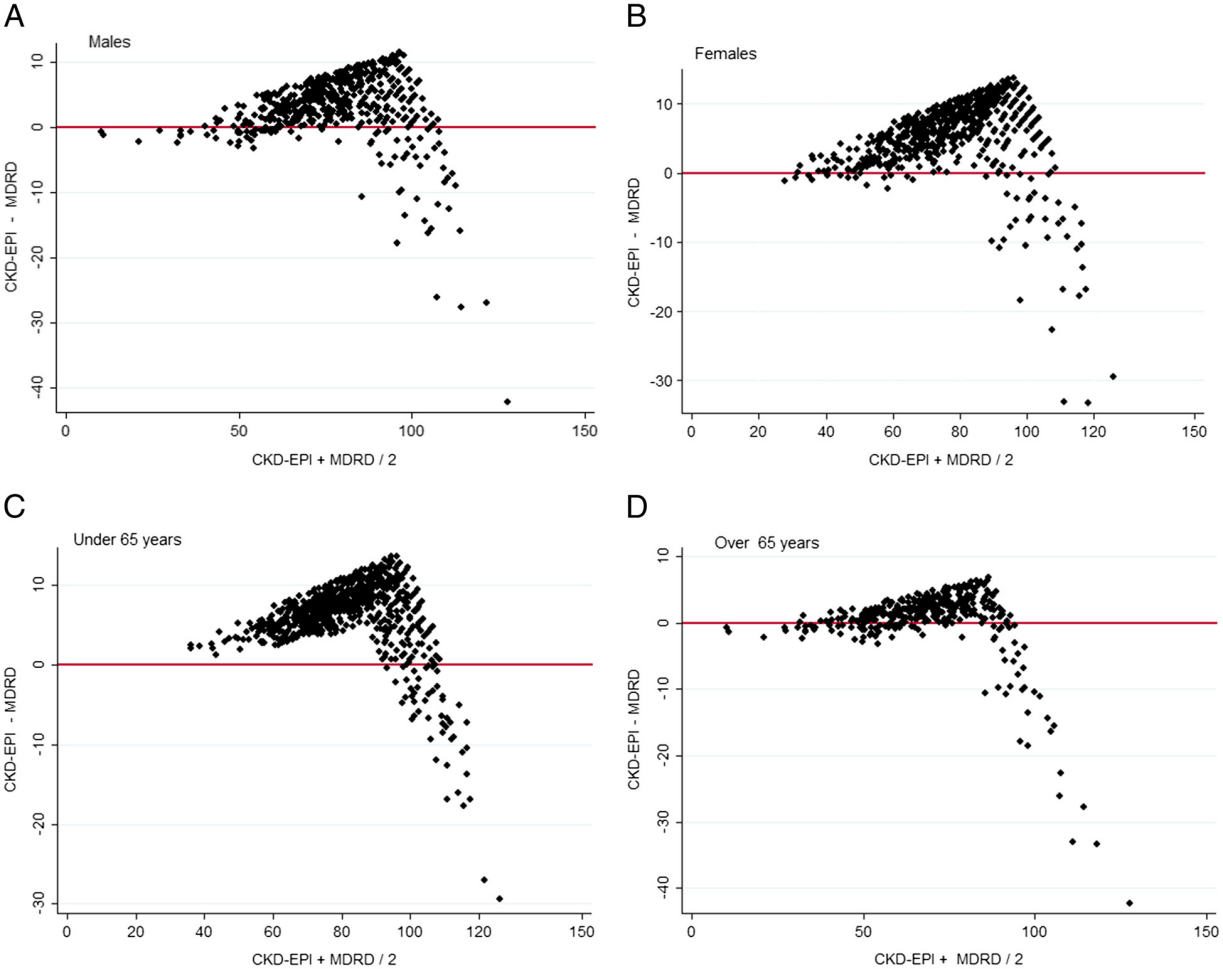
Bland Altman Plots (X axis mean CKD-EPI, MDRD, Y axis CKD-EPI − MDRD) in males.

**Figure 3:**
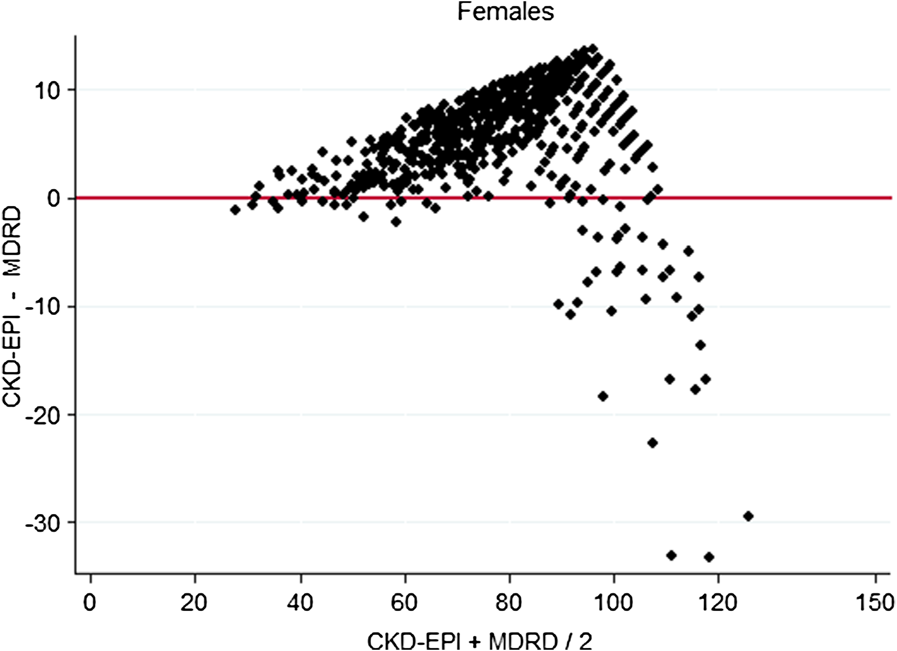
Bland Altman Plots (X axis mean CKD-EPI, MDRD, Y axis CKD-EPI − MDRD) in females.

**Figure 4:**
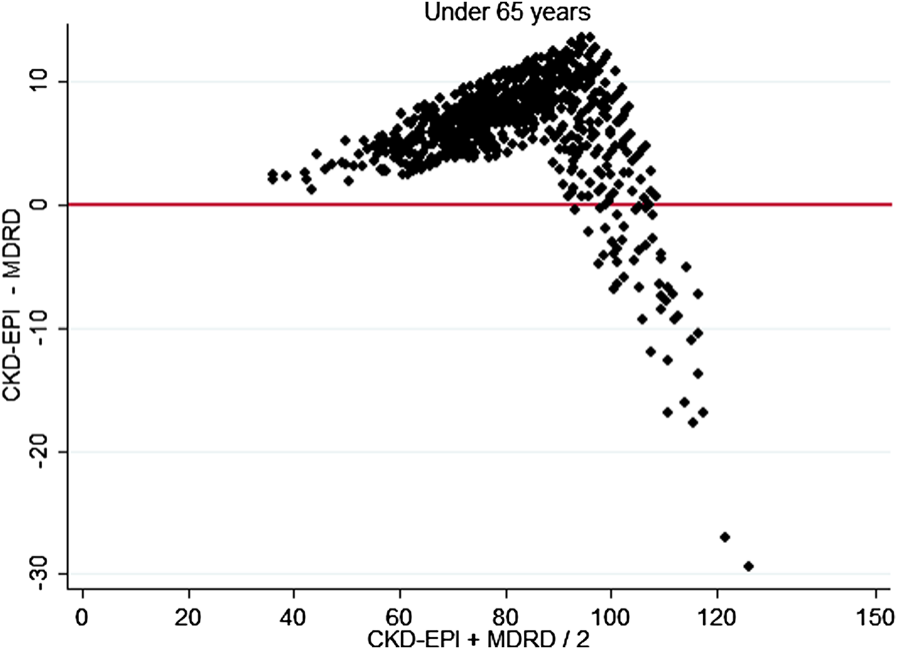
Bland Altman Plots (X axis mean CKD-EPI, MDRD, Y axis CKD-EPI − MDRD) in Subjects under 65 years.

**Figure 5:**
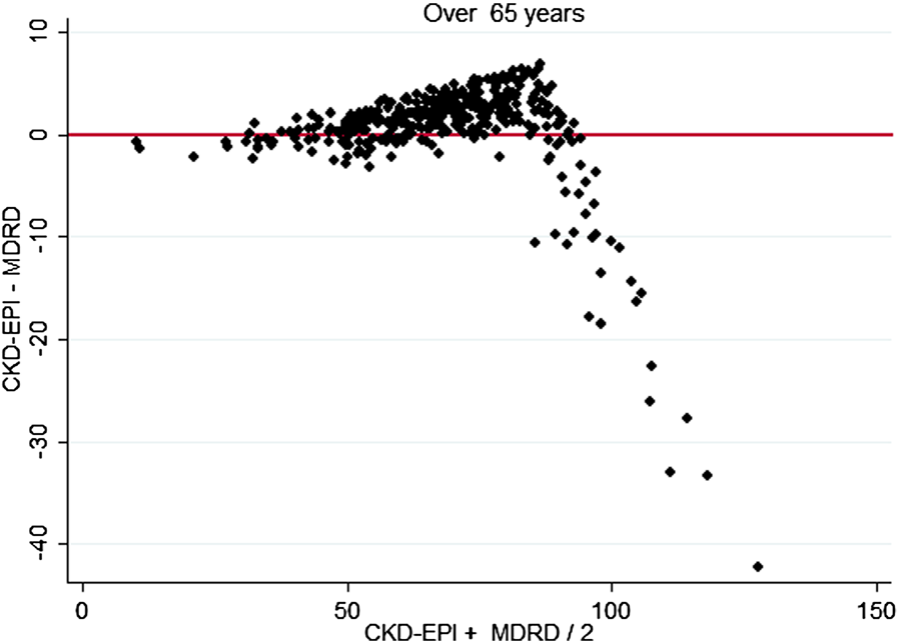
Bland Altman Plots (X axis mean CKD-EPI, MDRD, Y axis CKD-EPI − MDRD) in subjects over 65 years.

In comparing levels of bias between the methods, there was poorer agreement at higher GFR and better agreement between older aged subjects and males. These differences are demonstrated graphically by gender specific (Figures 2, 3) and age specific (Figures 4, 5) Bland Altman Plots [20]. A systematic bias is evident in all subjects, reporting lower values using the MDRD equation than CKD-EPI at average eGFR of up to approximately 100 mL/min, and thereafter for MDRD to estimate higher values than the CKD-EPI equation, with a greater bias in younger and female subjects.

### Associations of CKD with self rated health, education, and socio-economic group and estimated prevalent diabetics in this population

Poorer self rated health (p<0.001), lower socio economic status (p=0.006) and lower level education (p < 0.001) and diabetes (p < 0.03) were all associated with CKD (eGFR<60 mL/min/1.73 m^2^ or ≥60 with albuminuria), using a chi-squared test of association. Smoking and lack of physical exercise were associated with CKD but the association was not statistically significance. Moreover, in a logistic regression model adjusted for age and gender, lower level of education compared to higher level, OR (95% CI), 1.6 (1.1, 2.3), p=0.012 and poor self-rated health compared to excellent self rated health, OR: 1.6 (1.1, 2.2), p=0.02 continued to be significantly associated with CKD in this population (Table 4).

**Table 4:** Prevalence of CKD N % (CKD EPI <60 mls/min/1.73 m2 or Albuminuria>30 mg/g) by Self rated health Education Social Class and Diabetes Mellitus*, based on N=1098 (N=1095 for self rated health), OR (95% CI) adjusted for age and gender

**Covariate**	**Prevalence CKD**	**Chi**^**2**^**Test**	**OR (95% CI) Adjusted for age & gender**
	**Age Categories in Years**		***p<0.001***	p<0.001^*^
	45-54	48 (11.4%)		1.0
	55-64	46 (14.2%)		1.3 (0.84, 2.0)
	65-74	92 (34.2%)		4.2 (2.8,6.2)
	75+	55 (67.1%)		16.3 (9.4, 28.3)
	**Education Level**		***p<0.001***	p=0.04*
	Higher Level Education	64 (15.9%)		1.0
	Complete Secondary Level	43 (19.5%)		1.2 (0.76, 1.9)
	Complete Primary Education^§^	134 (28.2%)		1.6 (1.1, 2.3)
	**Self Rated Health**		***p<0.001***	***p=0.02***
	Excellent to Good Health	175 (19.4%)		1.0
	Fair to Poor Health	66 (34.4%)		1.6 (1.1, 2.2)
	**Social Class**		***p=0.006***	p=0.18*
	Social Class I &II	74 (17.4%)		1.0
	Social Class III & IV	84 (22.8%)		1.3 (0.92, 1.94)
	Social Class V & Unclassified	83 (27.2%)		1.4 (0.94, 2.0)
	**Diabetes Mellitus**		***p=0.027***	***p=0.17***
	**Diabetes Present**	27 (31.4%)^†^		1.44 (0.86,2.44)

* p value for non binary variables based on Likelihood Ratio Test of all levels compared to baseline.

^§^*Complete primary education includes incomplete secondary level education.*

^†^*31.4% (95% CI 21.4-41.4%) Diabetics had either proteinuria or GFR<60ml/min, 11.2% (95% CI 7.2-15.2%) of Diabetics had both Albuminuria and Impaired renal function.*

## Discussion

This is the first population based study of the prevalence of CKD in Ireland, using a randomly selected, nationally representative sample of middle and older aged adults and applying the recently published and more robustly derived CKD-EPI estimating equation. We found that approximately 1 in 8 persons in the Republic of Ireland have at least a moderate decrement in renal function (eGFR<60 mL/min/1.73 m^2^). As in previous studies, we find a marked age related increase in the estimated prevalence of renal dysfunction. The prevalence of eGFR<60 mL/min/1.73 m^2^ being 1.4% in those aged 45–50, all of whom had mild decrements in function and being over 25 fold higher (39.5%) in those aged over 70 years. As the age cut-off for entry into the SLAN physical examination study was age over 45 years, our population estimate only applies to this proportion of the population. However our age specific eGFR estimates are not dissimilar to NHANES 98–2006 estimates [21] reported using the CKD-EPI equation where for example 10.06% of subjects aged 60 to 69 had stage 3 CKD as had 35.33% of those aged over 70, the respective percentages for our study being 12.4% and 36.2%.

CKD-EPI equation was recently developed using pooled data to improve the accuracy of GFR estimates among individuals with normal or near normal renal function while remaining sensitive to the diagnosis of more serious renal impairment. As such it is more suitable for population-based studies. The MDRD equation reports lower levels of GFR in younger subjects, especially in females relative to the CKD EPI equation. The two methods were more concordant at lower eGFR and among older individuals. The CKD-EPI equation identified 33% of our study subjects as having normal renal function (G1), which is 50% more than that identified by the MDRD equation. Our results are broadly similar to that found in recent studies comparing MDRD and CKD-EPI estimates [22,23].

The overall population estimates are presented as weighted estimates to enable a population based eGFR, as weighting facilitates an unbiased estimate of survey based population statistics.

Estimation of GFR using regression equations is now routinely performed as part of a general biochemical screen, in many parts of the world. Using these equations for screening purposes without specific renal related indications may have important implications to an individual. The identification of mild but previously undiagnosed renal dysfunction in community based subjects is important, as these subjects may benefit from specific interventions, both in relation to renal disease and in cardiovascular risk attenuation. However, the misclassification of an individual without any evidence of renal damage as having Stage 2 rather than Stage 1 GFR, although not diagnostic by itself of CKD, may still have important implications in terms of self-perceived health status and with regard to medical insurability. Moreover, a recent large meta-analysis of population based cohort studies detected increased cardiovascular and all cause mortality in subjects with lower eGFR compared to Stage 1, even in the absence of albuminuria [24]. In current clinical practise in parts of Ireland, actual MDRD results for values up to 90 mL/min are reported, a policy also advocated by the 2007 revision of the Australasian Creatinine Consensus Working Group recommendations [25]. In view of the implications and the potential for misclassification using the MDRD equation at a eGFR of greater than 60 mL/min our results suggests that either the more precise CKD-EPI equation should be used for routine population screening or if the MDRD equation is to be used, the results should be right censored at a cut-off of 60 mL/min, below which the two methods show reasonable agreement.

In addition to GFR, albuminuria is also an independent predictor of end stage kidney disease [26], as well as all cause mortality and cardiovascular mortality. The detection and monitoring of albuminuria is thus also a potentially important screening test with broad implications. We detected albuminuria in 13.2% (95% CI 11.2, 15.2%), of the overall study sample, with 10.1% of those with eGFR≥60 mL/min having albuminuria thereby substantially increasing the proportion of subjects with eGFR≥60 mL/min who are defined as having CKD. The NHANES 99–2006 estimated albuminuria to be present in 7.3% of the non-institutionalized US population over 18 years of age with CKD-EPI eGFR ≥60 ml/min, while a recent study from Turkey [22], found 9.5% of such patients to be albuminuria. Some of these differences may related to secular trends of increased obesity in the Irish population, with the proportion of obese subjects (measured BMI >30 kg/m^2^) in Ireland increasing from 18% in 1999 [27] to 23% in the most recent survey [11].

CKD was associated in this study with poor self rated health and lower educational status and this reflects similar findings in other populations [8-10]. Education may be a more sensitive indicator of SES in this population. Age is a strong confounder being associated with CKD and poorer educational status which reflects the demographics of this middle and older aged Irish population. Although smoking and physical exercise were associated with CKD, neither reached significance in a univariate or multivariate model and may be a consequence of the cross-sectional design of the study.

Our study is limited, as in many of the available population based prevalence studies, we only have an available serum creatinine measurement from a single time point, although the confirmation of CKD requires that abnormalities are present over at least a 3 month interval. However, as nearly all the sample were community dwelling subjects in their baseline state of health and given our reasonable sample size it is likely that the mean estimates are reasonable representative of the population values. Our prevalence estimates are generally higher than other overall population estimates which relates to the increasing burden of chronic kidney disease with age, however they are similar to estimates in this age bracket. The response rate of 66% is also similar to other National Health and Lifestyle surveys [28] and is good for an unsolicited, relatively detailed survey in an era of longer working days, controlled access to housing and ever-increasing concerns over personal safety and the confidentiality of health related data. In comparison to the 2006 Republic of Ireland National Census, participants in our study were slightly over representative of women and higher socio economic group, as this may represent a greater willingness to undertake a physical examination and specimen collection. Urinary Albumin to Creatinine levels are higher than in NHANES study as they are based on one sample only in this study which may result in over estimation, however the higher estimates may also reflect the middle and older aged demography of the sample. Finally we have no actual gold-standard measurement of GFR, thus while we can compare results from the two estimating equations, we cannot examine the precision or bias of either estimate when compared with a gold standard. However both these equations are established in clinical practice, making population estimates important. In this Irish population the level of bias between the estimating equations especially at eGFR higher than 60 mL/min/1.73 m2 is similar to Stevens et al. in which the estimating equations were also compared to exogenous filtration markers [29].

## Conclusion

In conclusion, in a representative sample of middle and older aged Irish adults, using the CKD-EPI equation, we found that 11.8% (95% CI 9.9-13.8%) of subjects had an eGFR<60 mL/min/1.73 m^2^, with a similar weighted estimate of 11.6% (9.0%, 14.2%), to obtain unbiased population based statistics. 21.9% (19.5-24.4%) had either an eGFR<60 mL/min/1.73 m^2^ or eGFR≥ 60 mL/min/1.73 m^2^ in conjunction with albuminuria, with a weighted estimate of 21.3% (18%, 24.6%). The MDRD estimation equation which is in widespread use within Ireland, classified more subjects as eGFR<60 mL/min/1.73 m^2^ (14.9%, 12.8-17%), systematically underestimating eGFR at values below approximately 100 mL/min/1.73 m^2^ and overestimating it at higher values when compared to the CKD-EPI formula. The differences with the MDRD were most marked at eGFR greater than 60 mL/min/1.73 m^2^ for which specific values should not be reported.

## Competing interests

None declared. SLÁN 2007 was approved by the research ethics committee of the Royal College of Surgeons of Ireland. This study analyzed de-identified, secondary data and was therefore exempt from clinical research ethics committee review.

## Authors’ contributions

GB had a major role in data analysis and interpretation of results and drafted the paper. JL was part of the research team for the SLÁN 2007 and had a role in the statistical analysis and drafted part of the paper. JE contributed to the interpretation of results and revision of the paper. AF was involved in data analysis, interpretation as well as revisions to the paper. IP was a PI on SLÁN 2007 and made revisions to the paper. All authors approved the final version of the paper for publication. All authors read and approved the final manuscript.

## Pre-publication history

The pre-publication history for this paper can be accessed here:

http://www.biomedcentral.com/1471-2369/13/144/prepub
